# Shaping the future of tobacco through microbial insights: a review of advances and applications

**DOI:** 10.3389/fbioe.2025.1548323

**Published:** 2025-05-12

**Authors:** Wei Hu, Jiaxing Yuan, Jiaxiang Fei, Kaleem Imdad, Pengfei Yang, Shen Huang, Duobin Mao, Jing Yang

**Affiliations:** ^1^ College of Tobacco Science and Engineering, Zhengzhou University of Light Industry, Zhengzhou, China; ^2^ Technology Research Developing Center, Shenzhen Tobacco Industrial Co., Ltd., Shenzhen, China; ^3^ Department of Biosciences, COMSATS University Islamabad, Islamabad, Pakistan

**Keywords:** tobacco, microorganism, community composition, functions, isolation and cultivation strategies

## Abstract

Over the past 20 years, researchers have used multi-omics techniques to study microbial diversity and metabolic function on tobacco leaves. The unique metabolic function of tobacco microorganisms has attracted extensive attention from researchers, which is an important research field in tobacco industry to improve the intrinsic quality of tobacco leaf with microbial agents. The microorganisms are particularly rich on the surface of tobacco leaf, and their metabolic function is closely related to the change of tobacco leaf chemical composition. Some microorganisms have important metabolic functions, such as: degrading macromolecular and harmful substances in tobacco leaves, and they have different degradation rates and pathways for the substances. At present, many functions of tobacco leaf microorganisms have not been fully verified and analyzed. In the future, more novel culture methods are needed to screen and isolate microorganisms on the surface of tobacco leaves, deeply tap their metabolic potential, explore the application value of microorganisms in the tobacco industry, and further promote the innovation and development of the industry.

## 1 Introduction

**FIGURE 1:**
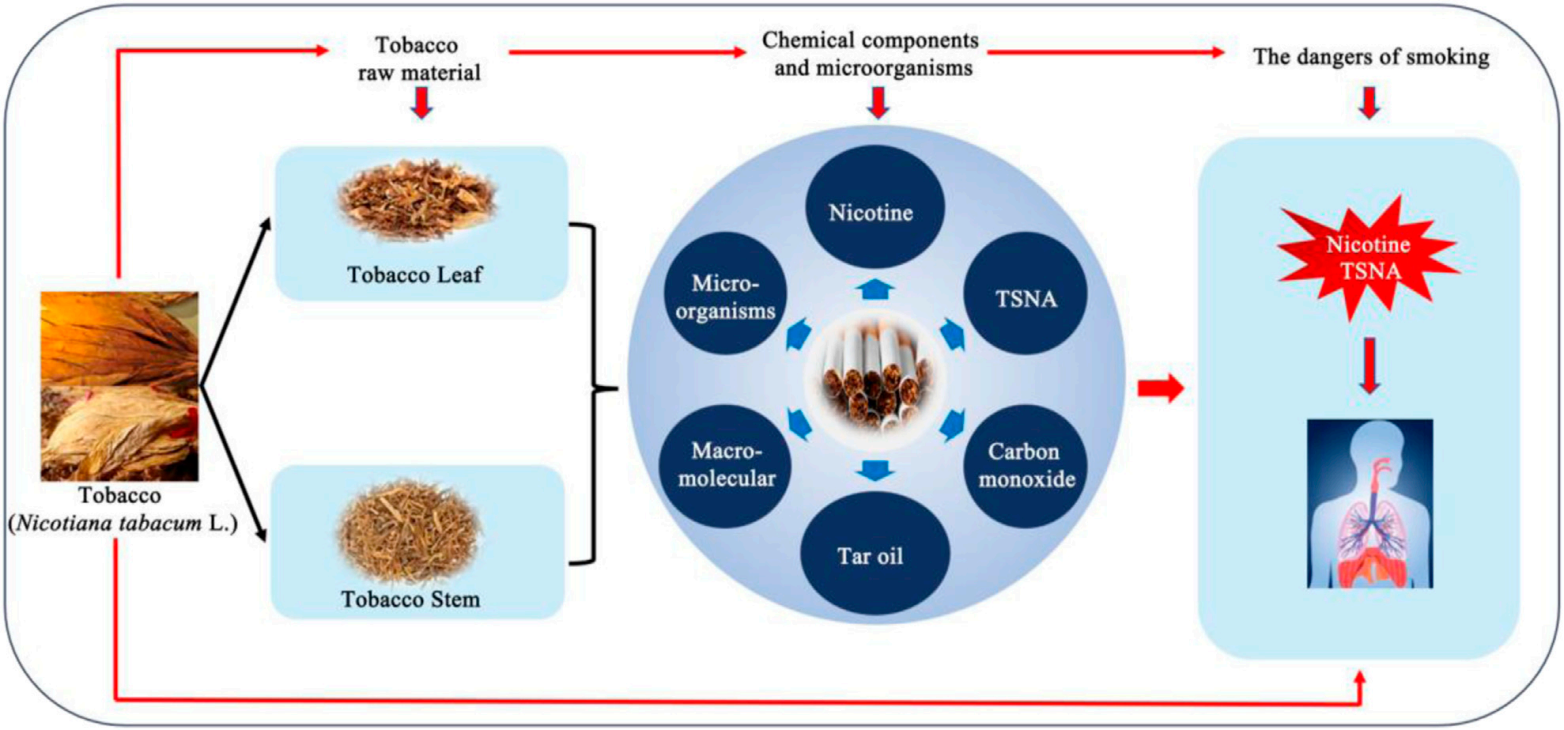
Human exposure to tobacco and its health outcomes.

Tobacco (*Nicotiana tabacum* L.) is an important agricultural nonfood crop and serves as the primary raw material for tobacco products (Zhang et al., 2020). Globally, China is the largest producer of tobacco where it is extensively cultivated in regions of South China, including Hainan and Sichuan (Xing et al., 2021). Tobacco products contain more than 6,000 chemicals, such as proteins, cellulose, starch, nicotine, tobacco-specific nitrosamines (TSNA) and numerous other toxicants (Chopyk et al., 2017b). It is well established that nicotine and TSNA, the predominant nitrogenous compounds in tobacco plants, play a crucial role in driving smoking addiction (Wang et al., 2018a) and pose a substantial threat to smokers’health (Chopyk et al., 2017b) (Figure 1).

The high concentration of starch, protein and other macromolecules causes an irritating burnt odor in tobacco leaves during combustion and reduce sensory quality. These macromolecules organic substances significantly affect the release of harmful substances in the flue gas during incomplete combustion (Yuan et al., 2006). Therefore, reducing the content of macromolecules and harmful substances in cigarette products can reduce the addiction and health hazards of cigarettes to smoking consumers (Figure 1). Previous studies have reported that the specific functional microorganisms are highly effective in improving the quality (Huang et al., 2022a). These microorganisms can enhance tobacco quality by improving aroma, degrading harmful substances, such as nicotine (Liu et al., 2015; Fitzpatrick, 2018; Xia et al., 2018; Mu et al., 2020; Zhang et al., 2022), TSNA (Wang and Huang, 2006; Shan et al., 2011; Jiang et al., 2021b), macromolecules (Li et al., 2006), and optimizing other desirable characteristics for smoking products during the aging process of tobacco leaves (Wang et al., 2018b).

Thus, the specific objectives of this review are: 1) to explore the community composition of tobacco microorganisms. 2) to outline the functions of tobacco microorganisms and discuss their biodegradation 3) to recap the isolation and culture techniques for tobacco-degrading microorganisms and recommend future research avenues in tobacco microbiology. This review provides an updated overview of the detailed description of microbial community composition on tobacco and its relationship with tobacco’s chemical composition, offering a more comprehensive and detailed analysis than previous reviews.

## 2 The structure and determinants of the tobacco microorganism community

### 2.1 The community composition of tobacco microorganisms

The microbial species identified through molecular techniques are abundant on tobacco leaves, in cigarettes, and across various smokeless tobacco brands (Chopyk et al., 2017b). It has been found that the number of culturable bacteria in tobacco leaves collected from plantations ranges between 2 × 10^3^ CFU/g and 7 × 10^5^ CFU/g, while those from manufacturing plants range between 2 × 10^3^ CFU/g and 8 × 10^3^ CFU/g (Larsson et al., 2008). Most existing research concentrating on the microbial diversity during different aging periods of tobacco leaves had been reported in the previous studies (Huang et al., 2010; Chen et al., 2018; Wang et al., 2018a; Zhang et al., 2020; Hu et al., 2021; Liu F. et al., 2021). High throughput sequencing analysis has revealed that the dominant bacterial communities on the surfaces of aging flue-cured tobacco belong to 48 genera, 36 families, and 7 phyla (Wang et al., 2018b). The predominant genera on flue-cured tobacco leaves include *Bacillus*, *Pseudomonas*, *Enterobacter*, *Sphingomonas*, *Pantoea* and *Methylobacterium* (Huang et al., 2010). And, cigar tobacco also exhibits a higher relative abundance of *Limnobacter*, *Brevundimonas*, unidentified_*Cyanobacteria* and *Pseudomonas*, with most of these species classified within just two bacterial phyla: Proteobacteria and Cyanobacteria (Xing et al., 2021). The core bacterial operational taxonomic units (OTUs) identified in cigarette tobacco comprise *Bacillus pumilus*, *Rhizobium* sp., *Sphingomonas* sp., unidentified members of *Enterobacteriaceae*, *Pantoea* sp., *Pseudomonas oryzihabitans* and *Pseudomonas putida* (Chopyk et al., 2017b). The bacterial communities in smokeless tobacco are primarily dominated by the phyla Firmicutes, Proteobacteria, Actinobacteria and Bacteroidetes (Han et al., 2016; Tyx et al., 2016; Smyth et al., 2019). Additionally, some bacterial endophytes have been identified in the leaves of tobacco, such as *Clostridium* sp. (Saito et al., 2008). At present, studies on the community composition of endophytic bacteria in tobacco leaf are very limited. Future studies need to further explore the diversity of endophytic bacteria in tobacco leaf and explore its importance in the process of tobacco leaf quality improvement.

Fungi are also an important group in the microbial composition of tobacco leaves (Chen et al., 2020). Studies have reported that the number of culturable fungi in plantation-grown tobacco leaves ranges from 0.3 × 10^3^ CFU/g to 3 × 10^3^ CFU/g and can reach up to 8 × 10^3^ CFU/g in the leaves collected from a manufacturing plants (Larsson et al., 2008). The diversity of fungi in the phyllosphere of tobacco leaves have been extensively studied using high throughput sequencing technologies (Lv et al., 2013). These studies revealed the existence of most abundant fungal genera including *Alternaria*, *Phoma*, *Cercospora*, *Aspergillus* and *Rhizopus* on tobacco leaves during curing (Chen et al., 2020). Whereas, genera such as *Cladosporium*, *Epicoccum*, *Trichoderma*, *Nigrospora*, *Penicillium*, *Chaetomium* and *Fusarium* are most frequently isolated and cultured from flue-cured and non-flue-cured tobacco leaves (Nagrale et al., 2016). Moreover, some fungal endophytes have also been found in the leaves, stems and roots of tobacco plants. At the phylum level, Ascomycota and Basidiomycota dominate fungal endophyte communities (Yuan et al., 2018; Jiang et al., 2021a). Genera such as *Altermaria*, *Apiotrichum*, *Cladosporium*, and *Microdium* are particularly abundant in both ordinary and “cherry-red” tobacco (Jiang et al., 2021a).

It is worth noting that tobacco-associated microorganisms also include potentially pathogenic species such as *P. putida* (Chopyk et al., 2017a; 2017b). And some fungi on tobacco leaves, such as *Alternaria* (Wang H. et al., 2016), *Phoma* *Rhizopus*, *Epicoccum* (Guo et al., 2020), are known plant pathogens. Up to now, the community composition of pathogenic microorganisms in tobacco microorganisms has not been reported, and the risk of ingestion and exposure to humans has not been analyzed and discussed in depth.

### 2.2 Factors influencing the community composition of tobacco microorganisms

The community composition of tobacco-associated microorganisms is dynamic, and influenced by various environmental factors, such as temperature, humidity and pH (Chopyk et al., 2017b; Chen et al., 2020). In bacterial community on the tobacco leaf of flue-curing procedure, the abundance of *Pantoea* and *Variovorax* is positively correlated with temperature and humidity, whereas the abundance of *Nesterenkonia*, *Staphylococcus*, *Chryseomonas*, *Rhodococcus*, *Paracoccus*, *Serratia* and *Ralstonia* shows a negative correlation with temperature and humidity (Hu et al., 2021). In the fungal community of the tobacco leaf phyllosphere during curing of leaves, the abundance of *Golovinomyces* is significantly affected by temperature, while the abundance of *Alternaria*, *Phoma*, *Trichoderma*, *Leptosphaerulina*, *Gibellulopsis* and *Candida* is notably impacted by relative humidity (Chen et al., 2020). One study also found that the fungal community diversity presented an obvious negative correlation with temperature and humidity during the flue-curing process (Hu et al., 2021). Furthermore, the community composition and diversity of tobacco-associated microorganisms are also influenced by fermentation duration (Liu F. et al., 2021). For example, the relative abundance of *Klebsiella variicola*, *Klebsiella pneumoniae*, *Serratia*, and *Salmonella* initially increases, peaking after 16 h before subsequently decreasing as the fermentation process continues to 24 and 36 h (Huang et al., 2024). Additionally, spatiotemporal variations affect the community composition of tobacco-associated microorganisms (Xing et al., 2021). For instance, the fungal community structure on the surface of tobacco leaves varies between different areas (Chen et al., 2018).

## 3 Functional roles of tobacco microorganisms

Tobacco microorganisms play crucial metabolic roles (Huang et al., 2024), which have a close connection with the chemical components of tobacco leaves (Liu F. et al., 2021). Previous study reported many attractive metabolic capacities in the aging flue-cured tobaccos microorganisms, including those involved in amino acid metabolism, carbohydrate metabolism, vitamin metabolism, the biosynthesis of flavors and fragrances, and the degradation of harmful compounds such as nicotine and nitrite (Wang et al., 2018b). During the aging process of tobacco leaves, the metabolic activities of microorganisms can consume protein and cellulose in tobacco leaves (Mou et al., 2020). At the same time, the macromolecular substances in tobacco leaf were decomposed into small molecular flavor substances such as viololanone, damalone and furfural (Mou et al., 2020). Moreover, it has been observed that the introduction of fungal agents during the aging of tobacco leaves can promote the co-regulation of chemical composition (Li et al., 2009) and facilitate the conversion of compounds within the leaves (Zheng et al., 2003).

Numerous reports have highlighted the crucial role of tobacco microorganisms in degrading macromolecular substances and harmful compounds (Chen et al., 2015; Liu F. et al., 2021; Huang et al., 2022a; 2022b), including the degradation of both types of hydrocarbons, i.e., aliphatic non-methane and aromatic compounds and other harmful substances (Liu F. et al., 2021). Several investigations have been published on the degradation of substances such as β-carotenes, starch, protein, phytosterols, as well as the harmful compounds like nicotine and TSNA (Table 1). For example, *Bacillus* species are known to degrade cellulose (Li et al., 2006), proteins (Chen et al., 2015), carotene (Huang et al., 2022a) and other compounds, thereby reducing irritation, bitterness, and astringency in tobacco during combustion. Consequently, several *Bacillus* species, including *Bacillus subtilis*, *Bacillus coagulans*, *Bacillus circulans*, *Bacillus megaterium* and *Bacillus thuringiensis*, have been employed to enhance the development of desirable aromas and improve the smoking qualities of tobacco (Zhao et al., 2007; Wang et al., 2018b). *Sphingomonas* sp. (Ma et al., 2016), *P. putida* HSM-C2 (Huang et al., 2022b), *Agrobaterium tumefaciens* sp. (Wang and Huang, 2006) have also been employed to degrade other substances, such as chlorogenic acid, coumarin, and TSNA. In addition, some fungi, including *Trametes versicolor* (Su et al., 2016), *Trametes hirsute* (Su et al., 2016), *Phanerochaete chrysosporium* (Su et al., 2016), *Moniliales Gliocephalias* sp. (Yang X. et al., 2014) have also shown effectiveness in degrading materials such as hemicellulose, and cellulose (Table 1).

**TABLE 1:** Summary of the tobacco components degradation by bacterial isolates and their culture media.

Strains	Medium	Substrate	Sources	Optimal conditions (pH and Tema)	Time	Degrading efficiency	References
Bacteria							
*Agrobacterium* sp. S33	Liquid minimal medium	Nicotine	Tobacco soil	pH 7; 30°C	6 h	100%	Wang S. N. et al. (2009)
*Acinetobacter* sp. ND12	Inorganic salt medium	Nicotine	Tobacco soil	pH 6; 28°C	11 h	90%	Li et al. (2011)
*Acinetobacter* sp. TW	Inorganic salt medium	Nicotine	Tobacco wastes	pH 7; 30°C	12 h	100%	Wang et al. (2011)
*Arthrobacter* sp. M2012083	—	Nicotine	Tobacco waste	—	—	—	Yao et al. (2012)
*Arthrobacter* sp. HF-2	Inorganic salt medium	Nicotine	Soil	pH 7.5; 30°C	43 h	100%	Ruan et al. (2006)
*Arthrobacter* sp. aRF-1	Inorganic salt medium	Nicotine	Soil	pH 7; 30°C	72 h	93.8%	Ruan et al. (2018)
*Bacterium* sp. J54	Liquid NIM medium	Nicotine	Tobacco leaf	30°C	54 h	85%	Jiang et al. (2021b)
*Ochrobactrum* sp. 4-40	Inorganic salt medium	Nicotine	Tobacco plantation soil	pH 7.0; 28°C	12 h	51.5%	Ma et al. (2012)
*Ochrobactrum intermedium* DN2	Utrient agar slants medium	Nicotine	Tobacco soil	pH 7.0; 30°C–37°C	24 h	93.4%	Yuan et al. (2007)
*Pseudomonas plecoglossicida* TND35	Nicotine inorganic medium	Nicotine	Tobacco soil	pH 7; 30°C	18 h	93.1%	Raman et al. (2014)
*Pseudoxanthomonas* sp. 5-52	Inorganic salt medium	Nicotine	Tobacco plantation soil	pH 7.0; 28°C	12 h	47.2%	Ma et al. (2012)
*Pseudomonas stutzeri* ZCJ	Inorganic salt medium	Nicotine	Tobacco leaf	pH 7.4; 37°C	24 h	1.5 g/L	Zhao et al. (2012)
*Pseudomonas* sp. ZUTSKD	Inorganic salt medium	Nicotine	Tobacco leaf	pH 7.0; 30°C	9 h	96.1%	Zhong et al. (2010)
*Pseudomonas* sp. HF-1	Inorganic salt medium	Nicotine	Tobacco waste soil	pH 7.0; 30°C	25 h	99.6%	Ruan et al. (2005)
*Pseudomonas* sp. Nic22	Inorganic salt medium	Nicotine	Tobacco soil	pH 6.5; 30°C–34°C	60 h	99.9%	Chen et al. (2008)
*Pseudomonas* sp.	Inorganic salt medium	Nicotine	Tobacco soil	pH 7; 30°C	10 h	100%	Wang et al. (2004)
*Pseudomonas putida*	Nicotine medium	Nicotine	Tobacco soil	pH 7; 30°C	12 h	100%	Wei et al. (2008), 2009
*Pseudomonas* sp. CS3	Mineral salt medium	Nicotine	Tobacco soil	pH 7; 30°C	24 h	98.6%	Wang et al. (2012)
*Pseudomonas* sp. S-1	Mineral salts medium	Nicotine	Tobacco powdery wastes	pH 7; 30°C	12 h	100%	Pan et al. (2018)
*Rhodococcus* sp. Y22	Nicotine selective medium	Nicotine	Tobacco soil	pH 7; 28°C	52 h	100%	Gong et al. (2009)
*Sinorhizobium* sp. 5-28	Inorganic salt medium	Nicotine	Tobacco plantation soil	pH 7.0; 28°C	12 h	72.5%	Ma et al. (2012)
*Sphingomonas* sp. TY	Inorganic salt medium	Nicotine	Tobacco wastes	pH 7; 30°C	18 h	100%	Wang et al. (2011)
*Saccharomyces cerevisiae* sp.	Enrichment medium	β-carotenes	Tobacco leaf	pH 8; 28°C	2 d	97.13%	Jia et al. (2015)
*Pseudomonas fluorescens* sp.	Enrichment medium	Nitrate	Tobacco leaf	pH 7.3; 30°C	10 d	68.77%	Shan et al. (2011)
*Pseudomonas fluorescens* sp.	Enrichment medium	Nitrite	Tobacco leaf	pH 7.3; 30°C	10 d	45.57%	Shan et al. (2011)
*Bacterium* sp. J54	Liquid NIM medium	Nitrosamines	Tobacco leaf	30°C	54 h	26.22%	Jiang et al. (2021b)
*Bacillus amyloliquefaciens* DA9	Liquid screening medium	Nitrosamines	Tobacco soil	—	45 d	47%	Wei et al. (2014)
*Sphingomonas* sp.	Liquid mineral medium	Chlorogenic acid	Tobacco leaf	pH 7.0; 37°C	6 h	100%	Ma et al. (2016)
*Pseudomonas putida* HSM-C2	Fermentation medium	Coumarin	Soil	pH 7; 30°C	24 h	99.83%	Huang et al. (2022b)
*Agrobaterium tumefaciens* sp.	Tryptic soy broth medium	TSNA	Tobacco leaf	—	45 d	81.32%	Wang and Huang (2006)
*Bacillus subtilis* FYZ1-3	Starch selective medium	Starch	Tobacco waste piles	—	—	—	Ye et al. (2023)
*Bacillus subtilis* FYZ1-3	Protein selective medium	Protein	Tobacco waste piles	—	—	—	Ye et al. (2023)
*Paenibacillus* sp.	Enrichment medium	Phytosterols	Tobacco leaf	pH 7.0; 37°C	50 h	38.5%	Ye et al. (2017)
Fungi							
*Aspergillus oryzae* 112822	Tobacco leaf extract medium	Nicotine	Tobacco leaf	pH 6.5; 28°C	40 h	2.19 g/L	Meng et al. (2010)
*Trametes versicolor*	—	Lignin	—	—	15 d	37.70%	Su et al. (2016)
*Trametes hirsute*	—	Lignin	—	—	15 d	51.56%	Su et al. (2016)
*Phanerochaete chrysosporium*	—	Lignin	—	—	15 d	53.75%	Su et al. (2016)
*Bacillus amyloliquefaciens* SL-7	Rescreening medium	Lignin	Tobacco straw	pH 7.0; 37°C	15 d	28.55%	Mei et al. (2020)
*Moniliales Gliocephalias* sp.	Enzyme-producing medium	Lignin	Soil	pH 6; 35°C	30 d	39.39%	Yang X. et al. (2014)
*Phanerochaete chrysosporium*	—	Hemicellulose	—	—	15 d	24.28%	Su et al. (2016)
*Trametes hirsute*	—	Cellulose	—	—	15 d	28.19%	Su et al. (2016)
*Moniliales Gliocephalias* sp.	Enzyme-producing medium	Cellulose	Soil	pH 6; 35°C	30 d	36%	Yang X. et al. (2014)

Furthermore, investigations into tobacco microorganism isolates have confirmed that certain bacteria exhibit a high degradation efficiency (>99%) for nicotine (Table 1). Notable examples include *Agrobacterium* sp. S33 (Wang S. N. et al., 2009), *Acinetobacter* sp. TW (Wang et al., 2011), *Pseudomonas* sp. Nic22 (Chen et al., 2008), *Pseudomonas* sp. (Wang et al., 2004), *P. putida* (Wei et al., 2008; 2009), *Rhodococcus* sp. Y22 (Gong et al., 2009), and *Sphingomonas* sp. TY (Wang et al., 2011). Therefore, it is of great scientific significance and practical value to search for functional strains with high degradation ability to nicotine to improve tobacco quality. However, some bacteria have low degradation efficiency, such as *Pseudoxanthomonas* sp. 5-52 (47.2%), *Ochrobactrum* sp. 4-40 (51.5%) and *Sinorhizobium* sp. 5-28 (72.5%) (Ma et al., 2012). Additionally, fungi also play an important role in the degradation of tobacco macromolecular substances, such as lignin and so on (Table 1), but the degradation efficiency of lignin is relatively low, such as *T. versicolor* (37.70%) (Su et al., 2016), *T. hirsute* (51.56%) (Su et al., 2016) and *Bacillus amyloliquefaciens* SL-7 (28.55%) (Mei et al., 2020). The different degradation efficiency may be related to the degradation characteristics of strains and culture conditions (Ruan et al., 2005; Gaekwad and Vinchurkar, 2018; Huang et al., 2022b). For instance, the degradation efficiency of nicotine was increased within the pH range (5.5–7.5) and decreased within the pH range (7.5–9.5) (Ruan et al., 2005). Ruan et al. found that the rising of temperature from 29°C to 41°C, could lead into profound decrease in the nicotine degradation (Ruan et al., 2005). While, Huang et al. revealed that variations in carbon or nitrogen source type, and ammonium nitrate contents cause a significant impact on the degradation rate of coumarin (Huang et al., 2022b).

The role of microorganisms in the fermentation process of tobacco leaves is closely related to the enzymatic reaction, as microorganisms secrete various enzymes (e.g., alpha-amylase (Ullah et al., 2021), protease (Yogesh and Halami, 2015), cellulase (Araújo et al., 2021) and so on) to the exocytosomes during their growth and development. These enzymes released into the extracellular environment catalyze the decomposition or synthesis of certain substances in tobacco leaves. Study reported that the neutral aroma-enhancing compound was positively correlated with the carbohydrate-active enzymes, such as glycoside hydrolase, glycosyltransferase, polysaccharide lyase, carbohydrate esterase, and auxiliary active enzyme (Huang et al., 2024). Based on the enzyme-producing characteristics of different microorganisms, some hybrid strains have also been utilized to optimize tobacco fermentation. For example, co-cultivation of *Bacillus amyloliquefaciens* LB with high alpha-amylase activity and *Bacillus kochii* SC with high neutral protease activity has been used to improve sensory quality of flue-cured tobacco (Wu et al., 2021). And the complementary culture of *Erwinia carotovora* could effectively degrade pectin and cellulose by producing pectin- and cellulose-degrading enzymes and then be used for the production of the neutral aroma-enhancing compound (Huang et al., 2024). Microorganisms on the surface of tobacco leaves produce not only xylanase, cellulase, pectinase, protease and amylase (Fan et al., 2013), but also nicotine-degrading enzymes (Table 2).

**TABLE 2:** The key enzymes and genes in the microorganisms for degradation of chemical components of tobacco.

Degrading substance	Strains	Enzyme/gene	References
Nicotine	*Arthrobacter* sp.	6-hydroxy-L-nicotine oxidase (6Hlno); 2,6-dihydroxypseudooxynicotine hydrolase (Ponh)	Huang et al. (2020)
	*Arthrobacter nitrophenolicus* ND6	2,6-dihydroxypyridine 3-hydroxylase (Dhph); nicotine dehydrogenase subunit (NdhA)	Wang et al. (2023)
		6-hydroxypseudooxynicotine dehydrogenase subunit (KdhL); nicotine blue oxidoreductase (NboR)	
		2-furoylCoA dehydrogenase (HmfB); (S)-6-hydroxynicotine oxidase (NctB); nicotine oxidoreductase (Nod); aerobic carbon-monoxide dehydrogenase (CodH); nicotinamidase (PncA); molybdenum cofactor cytidylyltransferase (MobA)	
Nicotine	*Pseudomonas* sp.	nicotine oxidoreductase (NicA); pseudooxynicotine amine oxidase (Pnao)	Huang et al. (2020)
	*Pseudomonas putida* S16	3-succinoylsemialdehyde-pyridne dehydrogenase (Sapd); 3-succinoylpyridine monooxygenase (SpmABC)	Xia et al. (2018)
	*Pseudomonas* sp. ZZ-5	6-hydroxy-3-succinoylpyridine hydroxylase (HspB); 2,5-dihydroxypyridine dioxygenase (Hpo)	Hu et al. (2019)
		N-formylmaleamate deformylase (Nfo); maleamate amidohydrolase (Ami); maleate cis/trans-isomerase (Iso); nicotinate hydroxylase (NicAB); 6-hydroxynicotinate monooxygenase (NicC)	Thisted et al. (2019)
		2,5-dihydroxypyridine dioxygenase (NicX); N-formylmaleamate deformylase (NicD); maleamate amidohydrolase (NicF); maleate cis/trans-isomerase (NicE)	Wei et al. (2017)
Nicotine	*Agrobacterium tumefaciens* S33	nicotine dehydrogenase (ndhAB); 6-hydroxynicotine oxidase (hno); aldehyde dehydrogenase (ald)	Huang et al. (2020)
	*Shinella* sp. HZN7	6-hydroxy-3-succinoyl-pyridine hydroxylase (hsh); 6-hydroxypseudooxynicotine dehydrogenase (pno)	
	*Ochrobactrum* sp. SJY1	N-formylmaleamate deformylase (nfo); maleate cis/trans-isomerase (Iso); maleamate amidohydrolase (Ami)	
Nicotine	*Bacillus subtilis* FYZ1-3	*nad*E, *gab*D, *yfk*N, *ppn*K, *pnc*C, *deo*D, *cca*, *pun*A, *nad*D, nadA, *nad*C, *nad*B, *ppn*K, *pnc*B	Ye et al. (2023)
Starch	*Bacillus subtilis* ZIM3	amylase amyE1	Dai et al. (2020)
Cellulose	*Bacillus subtilis* ZIM3	cellulase celE1	Dai et al. (2020)
Pectin	*Bacillus tequilensis* CAS-MEI-2-33	alkaline pectinase	Zhang et al. (2019)
Nitrite	*Debaryomyces hansenii* TOB-Y7	nitrite reductase (NiR)	Vigliotta et al. (2007)
Nitrite	*Pseudomonas putida* strain S16	*nic*A	Tang et al. (2009)
Xylan	*Bacillus methylotrophicus* sp.	xylanase	Fan et al. (2013)

**FIGURE 2:**
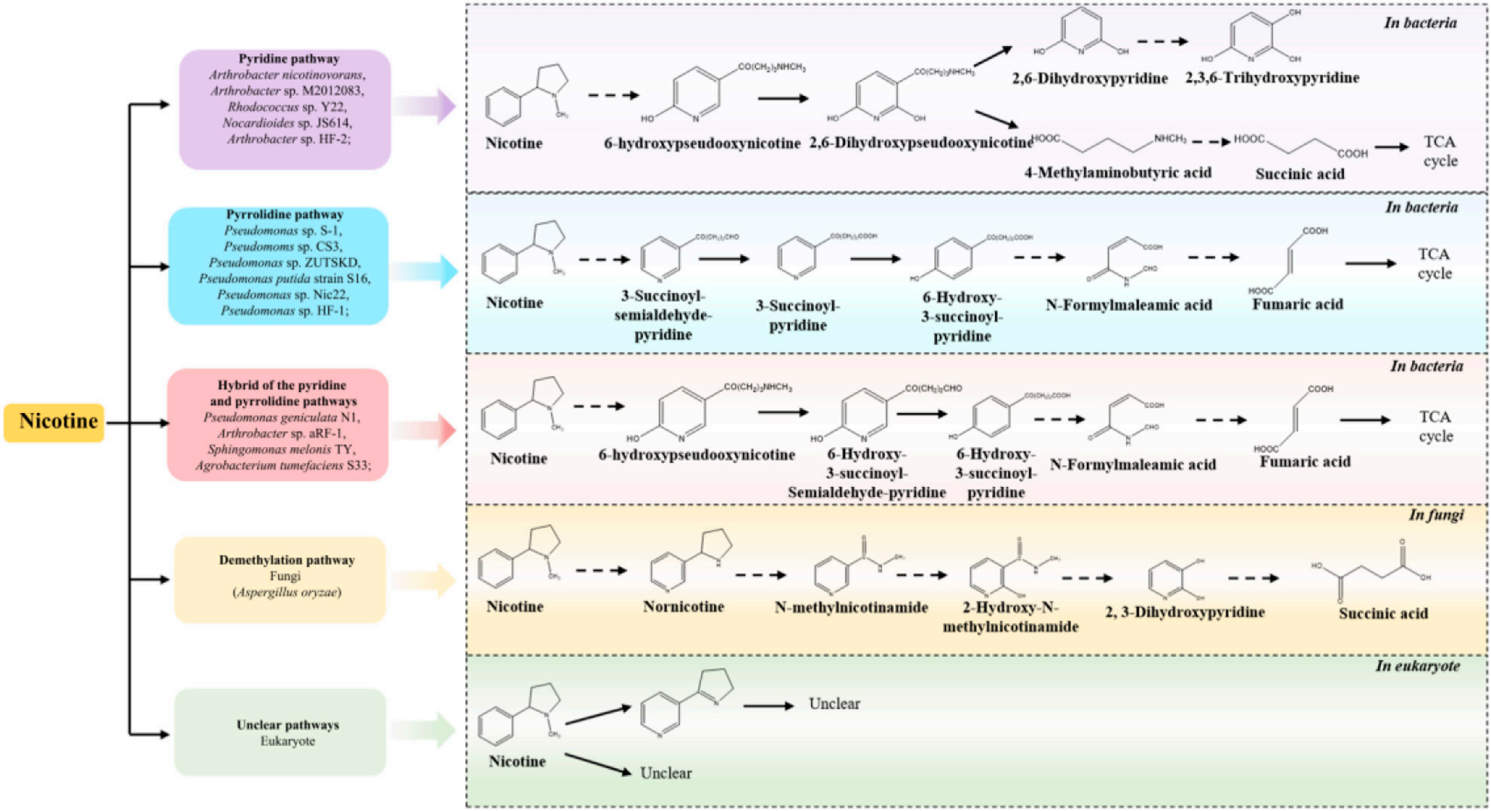
The degradation pathways of nicotine by *bacteria, fungi and eukaryotes* (adapted from Meng et al. (2010); Huang et al. (2020); Ganas et al. (2008); Ruan et al. (2006); Pan et al. (2018); Wang et al. (2012); Zhong et al. (2010); Tang et al. (2008); Chen et al. (2008); Ruan et al. (2006); Liu R. et al. (2021); Ruan et al. (2018); Wang et al. (2011); Wang S. N. et al. (2009)).

In recent years, the metabolism of harmful substances in prokaryotes, including the catabolic pathways for its degradation and the enzymes involved during the pathways, has been systemically investigated (Meng et al., 2010; Fitzpatrick, 2018; Huang et al., 2020), and nicotine metabolism is one of the most extensively studied pathways. (Fitzpatrick, 2018; Huang et al., 2020; Mu et al., 2020). In bacteria, three nicotine degradation pathways have been reported: the pyridine pathway, the pyrrolidine pathway and the hybrid Pathway (a combination of pyridine and pyrrolidine pathways, known as the VPP pathway) (Meng et al., 2010; Fitzpatrick, 2018; Huang et al., 2020). In fungi, such as *Aspergillus oryzae*, the demethylation pathway has been reported to be employed, while the pathways in the eukaryote remain unclear (Meng et al., 2010) (Figure 2). Key enzymes, such as Pnao, Pno and Ponh, have been identified as the representative enzymes in the pyrrolidine, pyridine, and VPP pathways, respectively (Table 2) (Mu et al., 2020). The methylamine from pseudooxynicotine and 6-hydroxypseudooxynicotine were removed by Pnao in the pyrrolidine pathway and Pno in the VPP pathway, while 2,6-dihydroxypseudooxynicotine was hydrolyzed to 2,6-dihydroxypyridine and 4-methylaminobutyrate by Ponh in the pyridine pathway (Mu et al., 2020). Additionally, amylase amyE1 and cellulase celE1 can be produced by *Bacillus subtilis* ZIM3, which can simultaneously degrade both starch and cellulose (Table 2) (Dai et al., 2020). Compared to nicotine, the metabolism of TSNA and macromolecule (such as starch, lignin and protein) has not been fully resolved in the strains (Table 1). Therefore, this situation limits the in-depth analysis to metabolism pathways and functions of TSNA and macromolecule in the environment.

Studies indicate that many bacteria possess all the essential genetic elements for nicotine catabolism (Mu et al., 2020). Nicotine-degrading genes annotated from the metagenome data, include *ndh*A, *nct*B, *kdh*L, *nbo*R, and *dhponh* are found to be actively involved in the pyridine pathway, which play an important roles in whole process of nicotine metabolism (Wang et al., 2023). Interestingly, *ndh*A and *nct*B are also the critical genes in the VPP pathway (Wang et al., 2023). Study reported that the *ndh*A gene is annotated to encode the isoquinoline 1-oxidoreductase alpha subunits, which can catalyze the hydroxylation of isoquinoline to 1-oxo-1,2-dihydroisoquinoline (Table 2) (Li et al., 2016). Additionally, genomic analysis of *Bacillus subtilis* FYZ1-3 revealed 14 functional genes associated with nicotine metabolism, and primarily located on the distinct genomic island of *Bacillus subtilis* FYZ1-3 (Ye et al., 2023). The homologous genes involved in nicotine catabolism, such as *cup*, *ponh*, *kdh*L, *TR*2, *kdh*M, *kdh*S, *nit*, *ndh*L, *ndh*S, *ndh*M, *cox*G, *dhph*, *pkc*, *mox*, *TR*1, *6hlno* have been identified in *Nocardioides* sp. JS614 and *Arthrobacter nicotinovorans* (Ganas et al., 2008). Furthermore, the genome of *Bacillus. subtilis* FYZ1-3 has been shown to harbor multiple metabolic pathways and numerous genes related to the degradation of carbohydrate and proteins. These include pathways for starch and sucrose metabolism (47 genes), glycolysis/gluconeogenesis (39 genes), amino sugar and nucleotide sugar metabolism (43 genes), biosynthesis of amino acids (122 genes), and alanine, aspartate and glutamate metabolism (33 genes) (Ye et al., 2023). In recent years, some organisms have been well utilized in the process of tobacco aging and fermentation, but most of the functions of microorganisms have not been developed and utilized. Therefore, developing more tobacco microbial resources and understanding their ecological functions have important guiding significance for the improvement of tobacco quality.

## 4 The application of functional microorganisms

Previous studies have reported that the addition of functional microorganisms can significantly improve the sensory quality of tobacco, such as *Arthrobacter* sp. (Xu et al., 2021), *Aureobasidium pullulans*, *Stenotrophomonas maltophilia*, H3-1. For example, the addition of functional strains (*Bacillus amyloliquefaciens* LB, *Bacillus kochii* SC and *Bacillus subtilis* subsp.) could promote an increase in aroma, softness and a decrease in irritation (Wu et al., 2021; Huang et al., 2022a). *Klebsiella variicola* H8 has the functions that increase neutral aroma-enhancing compound production, decrease the nicotine level and the water-soluble total sugar content in the reconstituted tobacco leaf concentrate solution (Huang et al., 2024). And the combination of aroma-producing yeast, *Lactobacillus debrueckii*, and *Rhizopus* had the most significant improvement in aroma, taste and smoke. Furthermore, some enzyme produced by microorganisms can significantly improve the quality of tobacco leaves, such as protease, amylozyme, pectinase, cellulase, which can catalyze the hydrolysis of corresponding substrates to produce flavoring substances and the precursors, so the corresponding catalytic hydrolysis mechanism has become one of the research hotspots in the tobacco industry.

## 5 Isolation and culturing strategies of tobacco microorganisms

**FIGURE 3:**
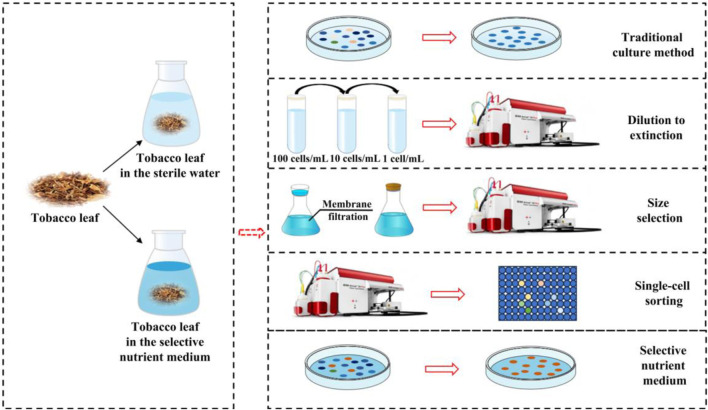
Isolation and culturing strategies and methods of tobacco’s microorganisms.

Traditional microbiological methods have played a crucial role in successfully isolating numerous microorganisms of interest and continue to be invaluable tools for cultivation (Lewis et al., 2021). During the isolation and cultivation of tobacco microorganisms, a variety of traditional techniques are employed (Figure 3). Common media, such as LB medium and inorganic salt medium, are typically used for this purpose (Chen et al., 2008; Mei et al., 2020). Additionally, selective nutrient media containing specific substrates, such as proteins, starch, and nicotine, are used to enrich specific microbial taxa (Raman et al., 2014; Ye et al., 2023). It is worth noting that successful isolation of strains using these approaches requires considerable time and patience, as well as meticulous optimization of media compositions and different physicochemical conditions (Lewis et al., 2021). Despite these refined efforts, the vast majority (>99%) of the microorganisms in the natural environment remained uncultured under laboratory conditions (Wang et al., 2021; Hu et al., 2022). To overcome the limitations of traditional culture methods, several innovative techniques have been developed to enhance microbial isolation and cultivation. For instance, single-cell sorting Via flow cytometry have been employed to isolate a greater diversity of strains from the tobacco microbial community, and two functional strains, *Bacillus amyloliquefaciens* LB (with high alpha-amylase activity) and *Bacillus kochii* SC (with high neutral protease activity) were successfully cultured (Wu et al., 2021). While other advanced techniques such as size selection, and dilution-to-extinction, have yet to demonstrate their universal applicability across different species and environments, they have already shown promise in culturing the marine bacteria (Figure 3) (Hu et al., 2022). For example, size selection, also referred as filtration, has been combined with flow cytometry to culture the small-sized bacteria (Hu et al., 2022). Similarly, dilution-to-extinction has been used to culture the marine bacterium, *Candidatus* Fonsibacter ubiquis LSUCC0530) in the previous study (Henson et al., 2018). In addition, bacteria with low nucleic acid content have been successfully cultured by using a combination of size selection, dilution-to-extinction and flow cytometry (Wang Y. et al., 2009), highlighting the potential and advantages of these key techniques in the isolation and culture of previously uncultured microorganisms (Wang Y. et al., 2009; Henson et al., 2018; Hu et al., 2022). In future studies, these innovative methods can be applied to the isolation and cultivation of uncultured tobacco microorganisms, paving the way for new discoveries and advancements in microbial research.

## 6 Challenges and future perspectives

Based on the above review and discussion, several challenges and perspectives should be considered in future research:

Although some tobacco microorganisms have been cultured, the number of strains capable of degrading large molecular substances and harmful compounds remains very limited. To date, only a few strains with a high efficiency in nicotine degradation have been reported. However, it is crucial to isolate or discover more functional strains, particularly those capable of degrading macromolecular substances and the harmful compounds. This will definitely develop a more precise understanding to accurately determine microbial characteristics including their metabolism and physiology as well as their ecological roles.

Although the previous studies have reported the intermediate degradation products and metabolic mechanisms of nicotine in the different strains, the comprehensive pathways, metabolic mechanisms and associated genes involved in the degradation of TSNA, β-carotenes, starch, protein and other compounds in tobacco microorganisms remain to be elucidated. In tobacco leaves, microorganisms are in a state of coexistence, displaying a competitive or symbiotic relationship with respect to substance utilization. Therefore, it is essential to conduct in-depth studies on the dynamic and long-term interactions between different microbial communities and their impact on improving tobacco quality.

Currently, the genetic mechanisms underlying the degradation of many tobacco-associated substances are not well understood. The integration of genomics, metagenomics, proteomics and systems biology represents a powerful approach to uncover the bacterial degradation mechanisms and provide valuable insights for further development of functional enzymes and genes. Additionally, advancing molecular biology techniques for the isolation and cultivation of functional tobacco microorganisms would be a crucial area for future research.

## 7 Conclusion

Tobacco microorganisms play a crucial role in enhancing tobacco quality, which represent a diverse group, primarily consisting of bacteria and fungi with marked metabolic capabilities, including amino acid metabolism, carbohydrate metabolism, vitamin metabolism, and the biosynthesis of flavors and fragrances. Additionally, these microorganisms secrete a variety of enzymes into the exocytosomes that can catalyze the decomposition or synthesis of certain substances in tobacco leaves. Tobacco microorganisms display remarkable degradation functions on the substances such as nicotine, TSNA, β-carotenes, starch, protein, and phytosterols, and contribute an important role in the enhancement of tobacco quality. Up to now, some organisms have been well utilized in the process of tobacco aging and fermentation, but most of the functions of microorganisms have not been explored and elucidated. Therefore, it is necessary to develop more microbial separation and culture methods for tobacco leaves, further explore the influence of microorganisms on the chemical composition of tobacco leaves, reveal the specific mechanism of their regulation and improvement of tobacco leaf quality, and provide a new scientific perspective and potential application path for the high-quality production of tobacco industry in the future.
